# Digital versus slit-beam marking for toric intraocular lenses in cataract surgery

**DOI:** 10.1186/s12886-022-02548-y

**Published:** 2022-07-27

**Authors:** Ning Ding, Xiaozhen Wang, Xudong Song

**Affiliations:** Beijing Tongren Eye Center, Beijing Tongren Hospital, Beijing Ophthalmology & Visual Science Key Laboratory, Capital Medical University, No.1 Dongjiaomin lane, Dongcheng District, Beijing, 100730 China

**Keywords:** Corneal astigmatism, Marking, IOL misalignment, Toric IOL, Callisto eye image-guided system, Cataract surgery

## Abstract

**Purpose:**

To compare the visual outcomes of digital and slit-beam manual marking for toric intraocular lenses (IOL) in cataract surgery.

**Setting:**

Single-center, Beijing Tongren Hospital, China.

**Design:**

Retrospective study.

**Methods:**

All patients with cataracts and regular corneal astigmatism greater than 0.75 diopters (D) underwent cataract surgery and astigmatism correction between June 2019 and June 2020. To mark the target axis of the toric IOL and the location of the incision, intraoperative digital marking was used by Callisto eye image-guided system in one group, while preoperative manual slit-beam marking was used in the other group. Uncorrected and best-corrected spectacle visual acuity, refraction, toric IOL axis, total higher order aberrations, coma, spherical aberration, and trefoil were evaluated at 1, 4, and 12 weeks postoperatively.

**Results:**

Seventy-two eyes of 58 patients were included. At 3 months after surgery, the mean residual refractive cylinder was 0.42 ± 0.45D in the digital group and 0.39 ± 0.40D in the manual group (*P* = 0.844). There were no significant differences between groups in spherical equivalent refraction, uncorrected and best-corrected spectacle visual acuity, or the parameters of vector analysis. All toric IOL alignment errors were within 10° of the intended axis, and among them, about 42% of eyes in the digital group and 61% of eyes in the manual group had a rotation of 0–2° (*P* = 0.038). Trefoil in the manual group decreased postoperatively compared with the digital group (*P* = 0.012). Other aberration analyses did not reveal any statistical differences between groups.

**Conclusions:**

Accurate slit-beam manual marking and digital image-guided marking are equally effective for toric IOL alignment.

## Introduction

As we all know, the accuracy of toric IOL alignment is crucial to the correction effect. Some experimental studies have found that the astigmatism correction will be significantly affected when toric IOL misalignment exceeds 10°, and a 45-degree misalignment may cause the total loss of toric correction and the image quality will be seriously reduced [1, 2]. An accurate marking method is important for aligning the final position of the toric IOL during surgery. Moreover, it is a controllable factor relative to toric IOL rotation and capsular bag shrinkage after surgery. There are two kinds of marking methods mainly used for toric IOL in cataract surgery in recent years, namely manual marking and digital-assisted marking. In the previous studies, the accuracy of digital marking was significantly better than that of manual marking [3–6], or the results of the two methods were similar [7, 8]. However, digital marking needs better equipment in the operating room, including the matching of the surgical microscope and digital image-guided system, which is difficult to provide for most small and medium-sized hospitals.

At present, there is no consistent conclusion on the postoperative refractive results using digital and manual marking for astigmatism correction. We retrospectively compared the refractive outcomes and visual quality of digital and slit-beam manual marking for toric IOLs in cataract surgery.

## Patients and methods

In this retrospective study, we included 72 eyes of 58 patients undergoing phacoemulsification and toric IOL implantation from June 2019 to June 2020 at Beijing Tongren Hospital. The study was approved by the Ethics Committee of Beijing Tongren Hospital, Capital Medical University (TRECKY2020-124) and conforms to the tenets of the Declaration of Helsinki (as revised in 2013).

Preoperative assessment included uncorrected distance visual acuities (UDVA), slit-lamp examination, and intraocular pressure, evaluation of IOL Master 700 (Carl Zeiss Meditec AG, Jena, Germany), pentacam HR (Oculus Optikgerate GmbH, Wetzlar, Germany), and OPD-scan III (Nidek Inc., Tokyo, Japan). Preoperative corneal astigmatism was determined by IOL Master 700. The inclusion criteria were as follows: cataract with regular corneal astigmatism > 0.75D, pupil dilation > 6.00 mm, and no obvious ocular or systemic diseases. The exclusion criteria were as follows: irregular corneal astigmatism, lens subluxation or pseudoexfoliation, uveitis, glaucoma, trauma, macular disease, retinopathy, or optic neuropathy.

All included patients underwent phacoemulsification and toric monofocal IOL implantation for astigmatism correction, including 36 eyes using digital marking by the Callisto eye image-guided system and 36 eyes using slit-beam manual marking. The power and orientation of the AcrySof Toric IOL (Alcon Laboratories, Inc., Fort Worth, TX, USA) were calculated by the Barrett toric calculator online (http://calc.apacrs.org/toric_calculator20/Toric%20Calculator.aspx), which took the preoperative biometric data and surgically induced astigmatism (calculated as 0.3) into account. A 2.4 mm clear corneal incision was used at a 160° axis. For the slit-beam manual marking group, a slit-lamp (SL-1E, Topcon Corporation, Japan) was used. The slit beam can be rotated to any angle from 0° to 180°, with a maximum length of 14 mm and a minimum scale of 5°. The patients were in a sitting position and kept their heads fixed. The slit lamp beam was centered on the patient's corneal apex and directed toward the target axis of the toric IOL and surgical incision, respectively (as determined by the calculation above). The marking was done on the corneal limbus using a 27-gauge needle and stained with a sterile pen under topical anesthesia with 2% proparacaine hydrochloride eyedrops. For the digital marking group, preoperative biometry data and intended axis were preset into the Callisto eye system (version 3.5.1.116555, Carl Zeiss Meditec AG). The intraoperative overlay was displayed under the OPMI Lumera 700 microscope (Carl Zeiss Meditec AG, Germany) to guide the surgeon in real-time. All surgeries were performed by the same experienced surgeon. No complications occurred.

Participants were followed up on 1 day, 1 week, 1 month, and 3 months postoperatively. Uncorrected and best-corrected spectacle visual acuity (BCSVA), manifest refraction, toric IOL axis, total higher order aberrations (HOA), coma, trefoil, and spherical aberration (SA) were recorded at each visit. The toric IOL axis was measured by the retro image using OPD-scan III.

The vector analysis for astigmatism correction was performed using the Alpins method [9, 10]. All statistical analyses were performed by SPSS software (version 22.0.0.0, IBM Corp., Armonk, NY, USA). Kolmogorov–Smirnov test was applied to check for normal distribution. T-test was used for comparison of the means between the groups. Chi-square(χ^2^) test was used to compare different percentages. A *P* value of less than 0.05 was considered statistically significant.

## Results

The demographic details and visual outcomes before and after surgery are shown in Table 1. The mean age of patients was 62.72 ± 15.96 (26 to 83) years in the digital group and 60.17 ± 16.26 (16 to 83) years in the manual group (*P* = 0.637). The mean preoperative corneal cylinder was 1.98 ± 0.74D (1.15 to 4.08D) in the digital group and 2.21 ± 1.01D (0.97 to 4.46D) in the manual group (*P* = 0.432). Astigmatism in both groups was mainly against the rule (ATR), followed by with the rule (WTR). At 3 months after surgery, the mean residual refractive cylinder was 0.42 ± 0.45D (0.00 to 1.25D) in the digital group and 0.39 ± 0.40D (0.00 to 1.25D) in the manual group (*P* = 0.844). The average UDVA was 0.15 ± 0.16 logarithm of the minimum angle of resolution (logMAR) (0.00 to 0.52 logMAR) in the digital group and 0.17 ± 0.12 logMAR (0.00 to 0.40 logMAR) in the manual group (*P* = 0.694, t-test of independent samples). The mean BCSVA was 0.04 ± 0.08 logMAR (-0.08 to 0.22 logMAR) in the digital group and 0.05 ± 0.07 logMAR (0.00 to 0.22 logMAR) in the manual group (*P* = 0.489, t-test of independent samples).

**Table 1:** Comparison of outcomes before and 3 months after surgery (mean ± SD)

	Digital Group	Manual Group	*P* value
Age (y) (range)	62.72 ± 15.96 (26 to 83)	60.17 ± 16.26 (16 to 83)	0.637
Gender (M/F)	11/17	8/22	-
Eyes (R/L)	16/20	17/19	-
Axial length (mm) (range)	24.18 ± 1.72 (20.9 to 26.4)	23.43 ± 1.33 (20.81 to 25.38)	0.153
Preop corneal cylinder (D) (range)	1.98 ± 0.74 (1.15 to 4.08)	2.21 ± 1.01 (0.97 to 4.46)	0.432
WTR	12	14	
ATR	23	22	
OB	1	0	
Residual refractive cylinder (D) (range)	0.42 ± 0.45 (0.00 to 1.25)	0.39 ± 0.40 (0.00 to 1.25)	0.844
Preop UDVA (logMAR) (range)	0.82 ± 0.39 (0.22 to 1.30)	0.72 ± 0.29 (0.4 to 1.30)	0.388
Postop UDVA (logMAR) (range)	0.15 ± 0.16 (0.00 to 0.52)	0.17 ± 0.12 (0.00 to 0.40)	0.694
Postop BCSVA (logMAR) (range)	0.04 ± 0.08 (-0.08 to 0.22)	0.05 ± 0.07 (0.00 to 0.22)	0.489

The clear corneal incision was made on the steep axis

*SD* standard deviation, *D* diopters, *Toric IOL* toric intraocular lens. *WTR* with the rule, *ATR* against the rule, *OB* Oblique, *UDVA* uncorrected distance visual acuity, *BCSVA* best-corrected spectacle visual acuity

**Fig. 1:**
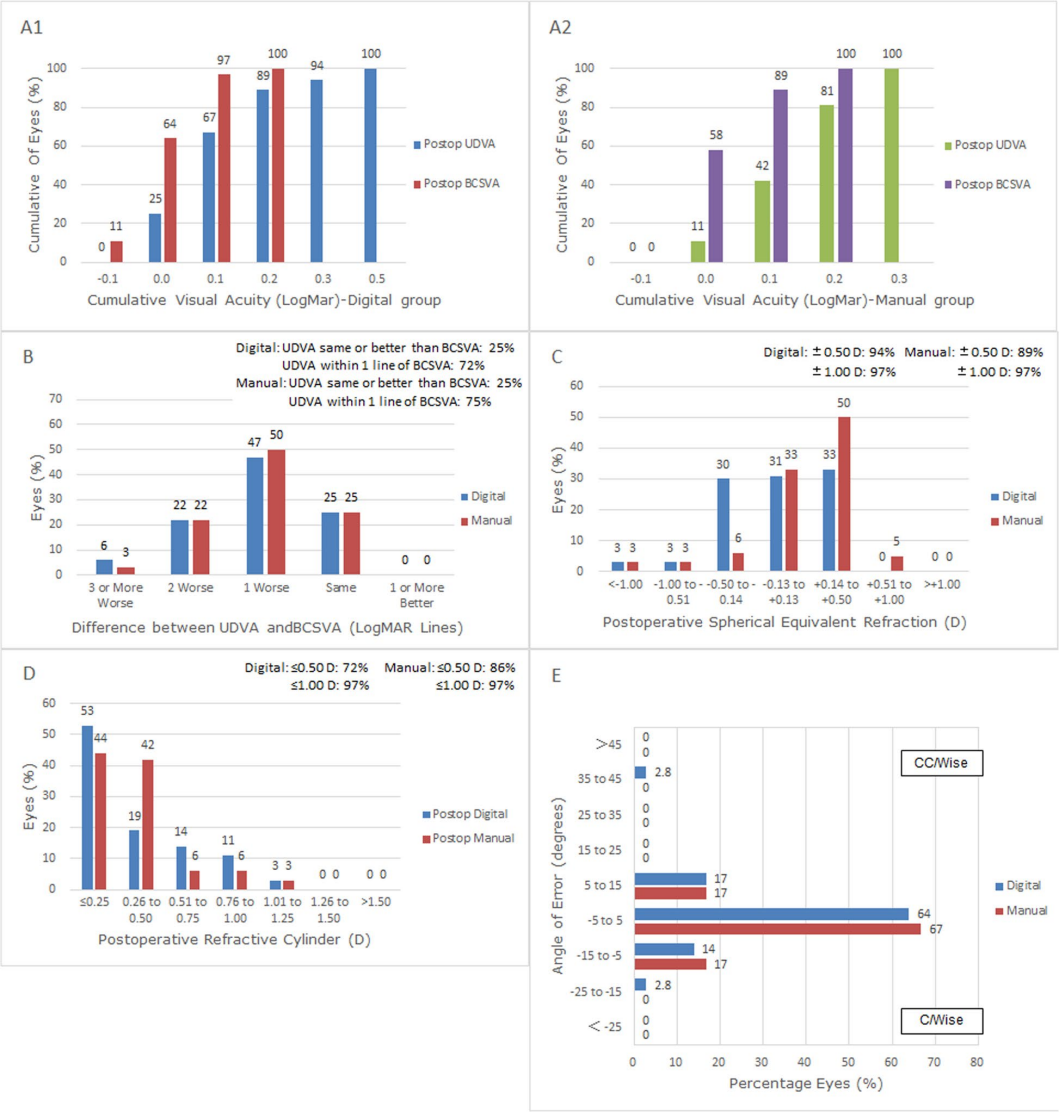
Comparison of refractive outcomes at 3 months postoperatively. **A** Uncorrected distance visual acuity. **B** Uncorrected distance visual acuity vs. best-corrected spectacle visual acuity. **C** Spherical equivalent refraction accuracy. **D** Postoperative refractive cylinder. **E** Refractive Astigmatism Angle of Error

The refractive outcomes at 3 months after surgery are shown in Fig. 1 by Standard Graphs for Cataract Surgery [10]. The percentages of postoperative UDVA and postoperative BCSVA were obviously increased in both groups. In digital cases with 0.2 logMAR, 89% of postoperative UDVA and 100% of postoperative BCSVA were obtained (Fig. 1 A1); in manual cases with 0.2 logMAR, 81% of postoperative UDVA and 100% of postoperative BCSVA were obtained (Fig. 1 A2). About 25% of eyes in both groups had the same lines in postoperative UDVA as BCSVA, while 72% in the digital group and 75% in the manual group were within 1 line of BCSVA (Fig. 1B). About 94% of digital cases and 89% of manual cases were within ± 0.50D in postoperative SE refraction (Fig. 1C). About 72% of digital cases and 86% of the manual cases were within ± 0.50D in the residual refractive cylinder (χ^2^ = 2.105, P = 0.147) (Fig. 1D). Angle-of-error analysis revealed that most eyes in both groups had an angle of error (AE) of between -15° and 15°. In the digital group, the arithmetic and absolute mean were 1.89° and 7.11° counterclockwise (CCW) respectively; in the manual group, they were 0.28° and 3.39° slightly CCW respectively (Fig. 1E, Table 2).

**Table 2:** Vector analysis for treatment and error at 3 months after surgery (mean ± SD)

	Digital Group	Manual Group	*P* value
TIA, D (range)	1.77 ± 0.78 (0.78 to 3.88)	2.01 ± 0.92 (0.96 to 4.20)	0.402
SIA, D (range)	1.80 ± 0.88 (0.40 to 3.88)	1.87 ± 1.03 (0.67 to 4.23)	0.832
DV, D (range)	0.41 ± 0.44 (0.00 to 1.23)	0.39 ± 0.39 (0.00 to 1.24)	0.883
AE, degrees			
arithmetic mean	1.89 ± 12.60	0.28 ± 5.29	0.620
(range)	(-22 to 39)	(-9 to 11)	
absolute mean	7.11 ± 10.44	3.39 ± 3.99	0.172
(range)	(0 to 39)	(0 to 11)	
ME, D (range)	0.03 ± 0.34 (-0.49 to 0.92)	-0.14 ± 0.37 (-1.01 to 0.49)	0.150
CI (range)	1.01 ± 0.23 (0.5 to 1.40)	0.91 ± 0.18 (0.6 to 1.21)	0.164
IOS (range)	0.31 ± 0.36 (0.00 to 1.26)	0.19 ± 0.18 (0.00 to 0.47)	0.235

*SD* standard deviation, *TIA* Target induced astigmatism, *SIA* Surgically induced astigmatism, *DV* Difference vector, *AE* angle of error, *ME* magnitude of error, *CI* correction index, *IOS* index of success

Table 2 shows the vector analysis results using the Alpins method. There were no significant differences in target induced astigmatism vector (TIA) (*P* = 0.402), surgical induced astigmatism vector (SIA) (*P* = 0.832) and angle of error, including arithmetic mean (*P* = 0.620) and absolute mean (*P* = 0.172) between the groups. The best result of magnitude of error (ME) is 0. The mean ME was 0.03 ± 0.34D in the digital group and -0.14 ± 0.37D in the manual group (*P* = 0.150). The correction index (CI) is preferably 1.0. The mean CI was 1.01 ± 0.23 in the digital group and 0.91 ± 0.18 in the manual group (*P* = 0.164). The best result for index of success (IOS) is 0. It was greater in the digital group (IOS = 0.31) than in the manual group (IOS = 0.19) (*P* = 0.235).

**Fig. 2:**
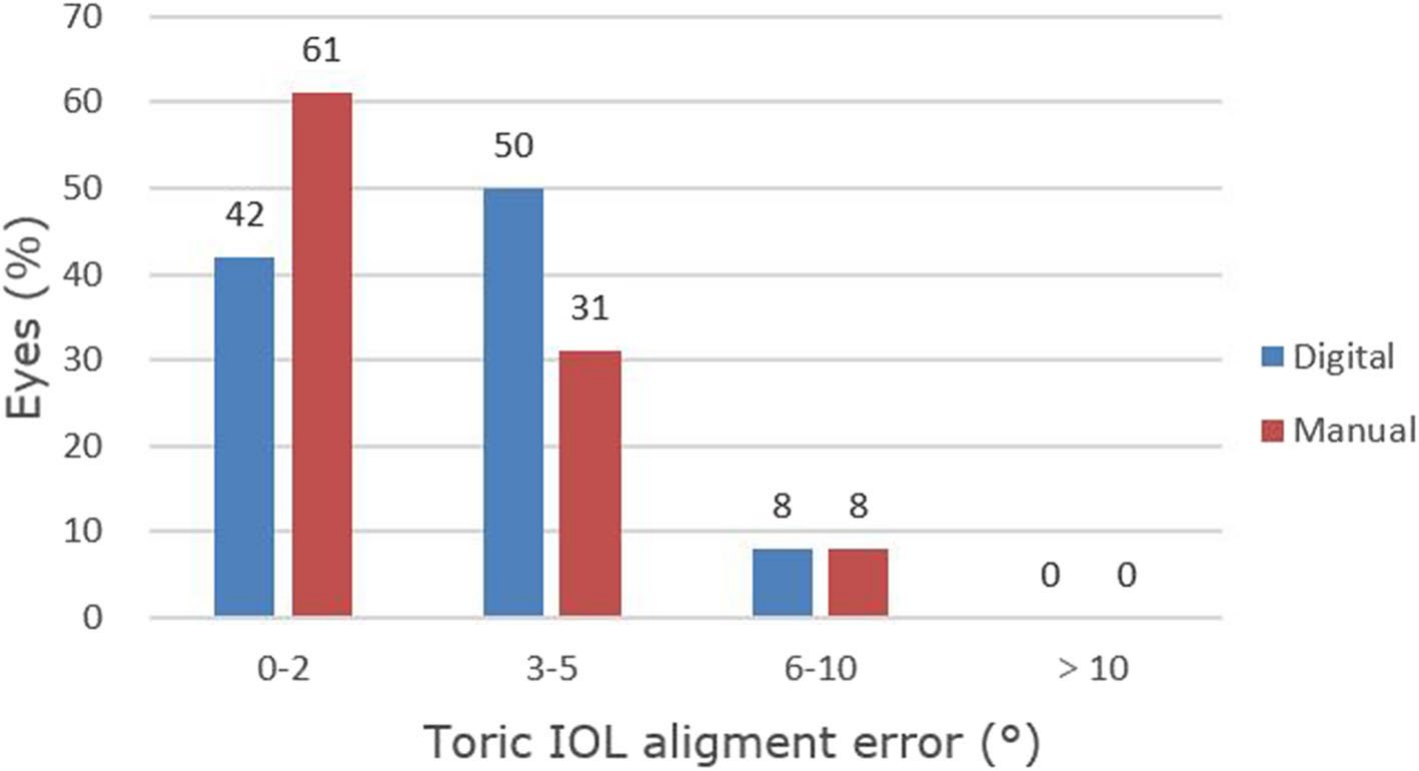
Between-group comparison of toric IOL alignment error

The toric IOL alignment error changes are summarized in Table 3, which is evaluated by OPD-scan III, including the changes of 1 day, 1 week, 1 month, and 3 months postoperatively. There were no significant differences between the two groups. Figure 2 shows the absolute difference in toric IOL alignment error in both groups 3 months after surgery. In absolute terms, all toric IOL misalignment was less than 10°. About 42% of eyes in the digital group had a rotation of 0–2°, compared with about 61% in the manual group (*χ*^*2*^ = 4.327, *P* = 0.038; Fig. 2). There was no significant difference between the two groups in other degrees. No eyes underwent secondary surgery to reorient the IOL. In this study, there was only one patient with an alignment error of 8° at 3 months postoperatively. He was a 78-year-old man in the digital group, with preoperative corneal astigmatism of 1.52D and a normal axis length of 23.59 mm. On the first day after surgery, the alignment error was 10°, visual acuity was 0.22 logMAR, and there was no discomfort. Therefore, no surgical repositioning was performed.

**Table 3:** Toric intraocular lens alignment error changes over time (degrees, Mean ± SD)

Change	Digital Group	Manual Group	*P* value
arithmetic mean			
1 day postop	-0.06 ± 4.61	1.11 ± 2.61	0.357
(range)	(-8 to 10)	(-3 to 5)	
1 week postop	-0.11 ± 4.13	1.00 ± 3.31	0.379
(range)	(-6 to 11)	(-5 to 6)	
1 month postop	0.39 ± 4.38	1.00 ± 2.11	0.598
(range)	(-6 to 12)	(-4 to 4)	
3 months postop	-0.94 ± 3.77	0.44 ± 3.07	0.234
(range)	(-6 to 8)	(-6 to 6)	
absolute mean			
1 day postop	3.50 ± 2.88	2.33 ± 1.53	0.141
(range)	(0 to 10)	(0 to 5)	
1 week postop	3.00 ± 2.74	2.78 ± 1.96	0.781
(range)	(0 to 11)	(0 to 6)	
1 month postop	3.17 ± 2.96	1.78 ± 1.48	0.083
(range)	(0 to 12)	(0 to 4)	
3 months postop	3.06 ± 2.29	2.33 ± 1.97	0.317
(range)	(0 to 8)	(0 to 6)	

*SD* standard deviation

The visual quality parameters, including total HOA, coma, trefoil, spherical aberration, and corneal HO root mean square (RMS) @4 mm, are summarized in Table 4. The total HOA, coma, and SA in both groups decreased after surgery, and the difference was statistically significant. Trefoil in the manual group also decreased postoperatively (*P* = 0.012). Besides, there was no significant difference in the other parameters above between the two groups.

**Table 4:** Preoperative and 3-month Postoperative aberration analysis (Mean ± SD)

Parameters	Digital Group	Manual Group	*P* value
Total HOA			
Preoperative	0.35 ± 0.22	0.44 ± 0.21	0.212
3-mo postoperative	0.24 ± 0.12	0.23 ± 0.12	0.826
*P*	0.041	0.001	
Coma			
Preoperative	0.11 ± 0.09	0.14 ± 0.07	0.208
3-mo postoperative	0.05 ± 0.02	0.09 ± 0.08	0.088
*P*	0.011	0.027	
Trefoil			
Preoperative	0.27 ± 0.21	0.33 ± 0.21	0.390
3-mo postoperative	0.21 ± 0.12	0.18 ± 0.12	0.478
*P*	0.237	0.012	
SA			
Preoperative	0.06 ± 0.05	0.09 ± 0.11	0.270
3-mo postoperative	0.03 ± 0.02	0.03 ± 0.02	0.956
*P*	0.019	0.020	
Cornea HO RMS@4 mm			
Preoperative	0.24 ± 0.35	0.20 ± 0.08	0.631
3-mo postoperative	0.19 ± 0.07	0.23 ± 0.12	0.351
*P*	0.566	0.453	

*SD* standard deviation, *HOA* Higher order aberrations, *SA* spherical aberration, *RMS* root mean square

## Discussion

In recent years, the widespread use of toric IOLs has brought good news to the cataract population with regular astigmatism and greatly improved independence on spectacles for them. At the same time, it also enhances the requirement for surgical precision. A retrospective analysis of 4949 eyes showed that the main source of error in toric IOL power calculation was preoperative corneal measurement (27%), followed by intraocular IOL misalignment (14.4%) and IOL tilt (11.3%) [11].

Accurate preoperative marking is crucial to reducing toric IOL misalignment. In the past, subjective direct visual marking or various markers were used for manual marking, such as bubble marker instrument [12], Mendez ring [7], and Pentium marker [13]. The patient needs to be kept in a stable sitting position. Generally, the horizontal meridian of 0–180° is identified first before the surgery, and then a second device is used to determine the toric IOL target axis according to the horizontal meridian during the operation, such as Mendez ring [14] or femtosecond laser [8]. However, manual marking has a troublesome procedure and requires a learning curve to achieve skill. Some thick marker pens make points broad or blurred, and the marks are easy to fade or even disappear during surgery. The line between marks on the eye may deviate from the center of the cornea, which may be an additional source of error in toric IOL imprecise alignment. In addition, manual marking is also affected by patient cooperation, which makes the accuracy of marking unstable.

Digital marking uses a markerless system. This method does not touch the patient's eyes during the whole process. It marks accurately and reduces the patient's psychological or eye discomfort. Moreover, it also prevents the errors associated with ink markers. Elhofi AH and Helaly HA compared the difference between VERION digital system and manual marking and found a lower deviation from the target induced astigmatism (TIA) and less postoperative toric IOL misalignment in the digital marking for toric IOL alignment [3]. Mayer WJ et al. also found better toric IOL alignment and significantly lower TIA in the digital marking group than in the manual marking group, which results in faster intraoperative IOL alignment and shorter overall surgical time [12]. Webers VSC et al. reported toric IOL misalignment was significantly less in the digital group than in the manual marking, but the study did not show significant advantages in terms of UDVA and residual refractive astigmatism using the digital marking system [4]. According to a meta-analysis, image-guided marking outperforms manual marking in terms of axis misalignment, difference vector, and postoperative astigmatism [5]. Titiyal et al. also observed significantly less misalignment with the Callisto eye system as compared with conventional manual marking with a bubble marker. Meanwhile, they found better visual quality in the image-guided surgery group and showed a higher internal Strehl ratio and MTF at all spatial frequencies [6]. However, digital image-guided marking requires a preoperative qualified anterior segment image, and intraoperative conjunctival edema or bleeding should be avoided to maintain a clear field of vision; otherwise, it may cause a matching error and result in misalignment.

Recent studies have shown that accurate manual marking and digital image-guided marking are equally effective in the alignment of toric IOL. Kodavoor SK et al. found that UDVA and CDVA were significantly improved in both manual and digital marking (VERION) groups. At three months after surgery, the UDVA and CDVA of the manual marking group were consistent with or better than those of the digital marking group [7]. Wu Q et al. measured the relative rotational deviation and vertical misalignment of the manually marked toric IOL and the incision axis, and the results showed that the marking deviation between the manual and the digital markers was small, and there was no significant difference between the two groups [15].

**Fig. 3:**
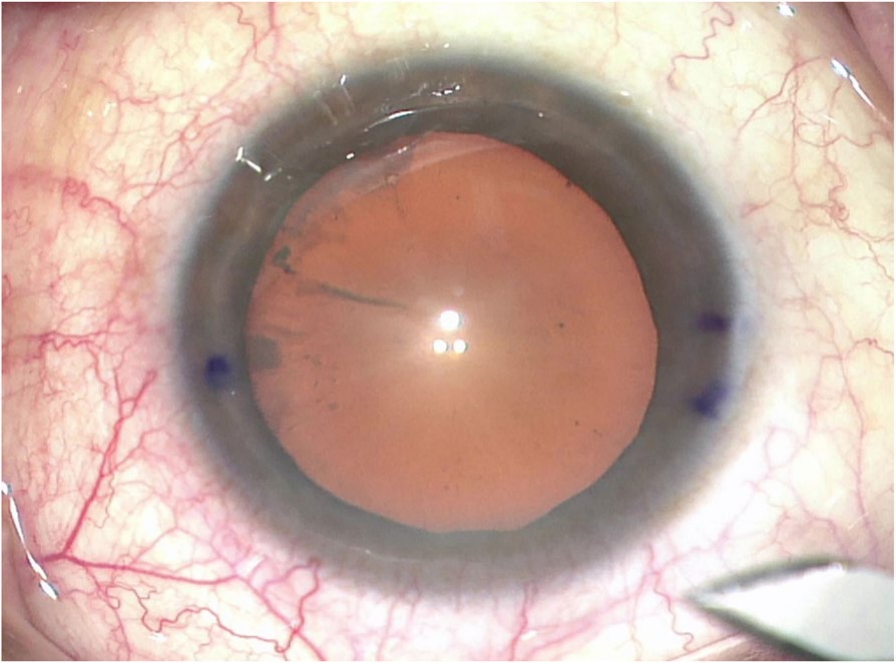
Manual marking. The blue dots represent the intended axis and incision

In the current study, with the patient in a sitting position and head stable, a 27G syringe needle was used to make a small break gently at the target position of the limbus. Then, stain the damaged epithelial surface with a fine-tip marker, which could make the ink markers more clear and long-lasting. In our marking procedure, the preoperative marking of the horizontal axis in the conventional method was canceled, and the target axis of the toric IOL and incision were directly marked according to the guidance of the slit-beam (Fig. 3). This approach optimizes the marking process and also helps the surgeon reduce the time and avoid deviations in manual marking. A second device is no longer needed to determine the toric IOL axis intraoperatively. Therefore, the time of toric IOL alignment was similar to that of digital image-guided system marking during surgery. In addition, only the superficial corneal epithelium was punctured during marking, and the injury was very minor. The epithelium healed completely and the cornea was clear on the first day postoperatively. No patients complained of obvious eye pain or discomfort, and there was no ocular surface infection.

At 3 months after surgery, the refractive outcomes showed that there was no significant difference between the two groups in postoperative residual astigmatism, spherical equivalent, UDVA, and BCSVA. There was also no significant difference between the digital group and the manual group in the percentage of the residual refractive cylinder within ± 0.50D, the mean absolute alignment error of the toric IOL axis, and the parameters of vector analysis. All toric IOL alignment errors are within 10° of the target axis. In particular, in toric IOL misalignment of 0–2°, the proportion of the manual group (61%) was higher than that of the digital group (42%), which was statistically significant (*P* = 0.038). Besides, the patient with an alignment error of 8 degrees was in the digital group. To our knowledge, the greatest IOL rotation occurred within 1 h after surgery and the toric IOL was highly stable after the first day postoperatively [16]. Therefore, the reason for the large degree of misalignment might be the toric IOL rotation in the early postoperative period, which was not relevant to the marking method. In vector analysis and postoperative visual quality, there was no significant difference in each parameter. The total HOA, coma, and SA of the two groups decreased after surgery, and the trefoil of the manual group also decreased (*P* = 0.012). The differences were statistically significant.

Compared with previous studies, we use a slit-beam and a fine needle to mark three points (two points for the axis of toric IOL and one for the incision). This marking method is simple and accurate and does not depend on expensive digital image-guided equipment. The method cancels the preoperative marking of the 0–180° horizontal reference axis and the second device during surgery, which could avoid associated alignment errors and greatly improve the postoperative visual quality of patients. However, the accuracy of manual marking depends on the good cooperation of patients and the high proficiency of surgeons. Owing to the learning curve of manual marking, the accuracy can vary among different surgeons. The more experienced the operator manually marks, the more accurate toric IOL alignment results are likely to be.

In conclusion, both the slit-beam manual marking and the digital image-guided system marking method are accurate for toric IOL alignment, and the two methods can be replaced with each other. Manual marking is a good supplementary marking method when the IOL master 700 cannot take a qualified image of the anterior segment. The precise application of toric IOL should not be limited by the digital facilities, especially in small peripheral centers. Due to the influence of COVID-19, long-term observation is lacking in our study. Further studies of long-term follow-up with a larger sample size are required to assess the accuracy of these marking techniques.

## Data Availability

The datasets for the study are not public due to personal privacy but are available from the corresponding author on reasonable request.
